# Constructing immune and prognostic features associated with ADCP in hepatocellular carcinoma and pan-cancer based on scRNA-seq and bulk RNA-seq

**DOI:** 10.3389/fimmu.2024.1397541

**Published:** 2024-05-07

**Authors:** Zhengwei Zhang, Yuying Li, Zhen Quan, Yapeng Li, Liying Zhu, Shibo Sun, Xiaoning Chen

**Affiliations:** ^1^ Department of General Surgery, The Second Affiliated Hospital of Harbin Medical University, Harbin, China; ^2^ Department of Infectious Diseases, The Second Affiliated Hospital of Harbin Medical University, Harbin, China; ^3^ Department of Critical Care Medicine, The Second Affiliated Hospital of Harbin Medical University, Harbin, China

**Keywords:** antibody-dependent cellular phagocytosis, hepatocellular carcinoma, prognosis, immunity, pan-cancer

## Abstract

**Aim:**

Despite the significant therapeutic outcomes achieved in systemic treatments for liver hepatocellular carcinoma (LIHC), it is an objective reality that only a low proportion of patients exhibit an improved objective response rate (ORR) to current immunotherapies. Antibody-dependent cellular phagocytosis (ADCP) immunotherapy is considered the new engine for precision immunotherapy. Based on this, we aim to develop an ADCP-based LIHC risk stratification system and screen for relevant targets.

**Method:**

Utilizing a combination of single-cell RNA sequencing (scRNA-seq) and bulk RNA-seq data, we screened for ADCP modulating factors in LIHC and identified differentially expressed genes along with their involved functional pathways. A risk scoring model was established by identifying ADCP-related genes with prognostic value through LASSO Cox regression analysis. The risk scoring model was then subjected to evaluations of immune infiltration and immunotherapy relevance, with pan-cancer analysis and *in vitro* experimental studies conducted on key targets.

**Results:**

Building on the research by Kamber RA et al., we identified GYPA, CLDN18, and IRX5 as potential key target genes regulating ADCP in LIHC. These genes demonstrated significant correlations with immune infiltration cells, such as M1-type macrophages, and the effectiveness of immunotherapy in LIHC, as well as a close association with clinical pathological staging and patient prognosis. Pan-cancer analysis revealed that CLDN18 was prognostically and immunologically relevant across multiple types of cancer. Validation through tissue and cell samples confirmed that GYPA and CLDN18 were upregulated in liver cancer tissues and cells. Furthermore, *in vitro* knockdown of CLDN18 inhibited the malignancy capabilities of liver cancer cells.

**Conclusion:**

We have identified an ADCP signature in LIHC comprising three genes. Analysis based on a risk scoring model derived from these three genes, coupled with subsequent experimental validation, confirmed the pivotal role of M1-type macrophages in ADCP within LIHC, establishing CLDN18 as a critical ADCP regulatory target in LIHC.

## Introduction

According to the latest data from GLOBOCAN, conducted by the International Agency for Research on Cancer (IARC), liver cancer ranks sixth among cancers globally and is among the top three cancer-related causes of death (1). Notably, when separating the statistics for the two primary histological subtypes of primary liver cancer, the proportion of intrahepatic cholangiocarcinoma (iCCA) is relatively small, whereas liver hepatocellular carcinoma (LIHC) accounts for over 80% of all liver cancer cases. The latter is among the top three causes of cancer-related deaths in 46 countries and ranks within the top five in 90 countries (2). Despite the opportunity for surgical treatment in early-stage liver cancer patients, the majority are unfortunately diagnosed at advanced stages of the disease, facing poor outcomes with postoperative recurrence and metastasis (3). For patients with advanced liver cancer, systemic treatment becomes particularly crucial. Approximately 50-60% of liver cancer patients undergo systemic therapy, half of whom are initially diagnosed with advanced stage LIHC, while the other half receive treatment following the progression of LIHC (4).

LIHC is a malignant tumor characterized by a highly immunosuppressive microenvironment, rendering immunotherapy a promising treatment strategy (5). Antibody-based immunotherapy holds a pivotal position in the realm of cancer immunotherapy, especially in the treatment of specific cancer types. For instance, the combination of atezolizumab and bevacizumab (6) as a first-line treatment for liver cancer, the pairing of tremelimumab with durvalumab (7), and the second-line treatments involving durvalumab (8), as well as the U.S. approved alternative second-line options for patients initially treated with sorafenib, including pembrolizumab or nivolumab in conjunction with ipilimumab (9, 10), are all examples of antibody-based immunotherapy. However, despite significant therapeutic advancements in systemic treatments, only ≤30% of patients show an improved objective response rate (ORR) to the current standard treatments (4). Therefore, the development of more effective immunotherapy protocols is imperative.

Antibody-dependent cellular phagocytosis (ADCP) immunotherapy is regarded as a new engine for precision immune treatment, involving the identification and marking of cancer cells by antibodies, followed by the recognition of these marked targets by phagocytic cells such as macrophages, leading to their phagocytosis. This approach has proven effective in the treatment of most tumors (11). Kamber RA and colleagues, through a comprehensive genome-wide CRISPR knockout overexpression screening platform, had discovered numerous ADCP regulatory factors and identified a set of genes that impeded antibody-dependent cellular phagocytosis (12). To date, no therapies that explicitly mediate ADCP have been established, but related research has begun to emerge. Arulanandam A and others had demonstrated the efficacy of CYT-303, designed against GPC3, in triggering ADCP in hepatocellular carcinoma cell lines (13). Similarly, Chen Y and associates had developed OBI-888, targeting Globo H, which had triggered antibody-dependent cell-mediated cytotoxicity and ADCP in various xenograft cancer models, including liver cancer, inhibiting tumor growth (14). These findings underscore the potential value of ADCP immunotherapy.

In our study, we integrated the use of scRNA-seq and Bulk RNA-seq data to screen for ADCP regulatory factors identified in the research by Kamber RA et al. within the context of LIHC. This enabled the identification of differentially expressed genes and their potential functional pathways. Through LASSO Cox regression analysis, ADCP-related genes with prognostic value were identified to establish a risk scoring model. Additionally, assessments related to immune infiltration and immunotherapy were conducted on the risk scoring model, along with pan-cancer analyses of key target. Our findings unveiled several critical ADCP-related genes involved in the progression of LIHC.

## Methods

### Data collection

RNA-seq data and clinical information for LIHC were downloaded from The Cancer Genome Atlas (TCGA) database. This dataset includes samples from 374 LIHC tumors and 50 normal liver tissues. Patient information typically includes, but is not limited to, the patient’s age, gender, pathological stage, treatment history, survival time, and survival status. It is noted that within the database, patient data for hepatocellular carcinoma exhibiting missing values in various clinicopathological characteristics at follow-up and cases with incomplete clinicopathological data were excluded from analyses related to clinical correlations. Single-cell datasets were obtained from the Gene Expression Omnibus (GEO) website, under the accession number GSE149614. The gene set related to ADCP was derived from PMID: 34497417, encompassing a total of 620 genes.

### Single-cell data quality control

Single-cell data quality control was conducted using the LIHC single-cell dataset generated with the Cell Ranger software package, leading to the creation of a Seurat object that included a gene expression matrix and sample annotation information. Subsequent single-cell analyses were performed using Seurat v3.1.4. During the quality control phase, cells with gene counts between 100 and 6000, unique molecular identifier (UMI) counts greater than 200, and mitochondrial gene expression below 10% were retained. Setting a lower limit for gene counts is intended to exclude low-quality cells with minimal gene expression data, while establishing an upper limit is aimed at excluding situations involving doublets or clusters of multiple cells. Setting a minimum value for UMI counts ensures that the selected cells contain sufficient RNA molecules, thereby reflecting the true biological state of the cells. Standard Seurat procedures were followed, encompassing normalization, identification of highly variable genes, scaling, principal component analysis, and batch effect correction using Harmony. Cells achieving an accumulated variance of 80% were preserved for further clustering analysis.

### Cluster analysis and cell annotation

Clustering was performed at the optimal resolution value identified through t-SNE visualization. To enhance the accuracy of cell annotation, samples derived from both tumor and normal tissues were not distinguished at this stage. Cell subtypes were annotated based on their molecular expression patterns. The exploration of differentially expressed genes between subtypes and groups was conducted using the FindAllMarkers function in Seurat, employing the Wilcoxon test as the statistical method with default parameters.

### Construction of clinical prognosis model

Initially, the intersection of differentially expressed genes and ADCP-related genes was identified. Subsequently, the R caret package was employed to perform cross-validation on the gene expression matrix, which possessed complete clinical information, segregating the dataset into a training set (train) and a test set (test) in a random 0.7:0.3 ratio. The training set was utilized to construct a risk prognosis model for LIHC, while the test set served to evaluate the model’s performance. Utilizing the prognosis model based on ADCP-related genes, risk scores for 424 LIHC samples from the TCGA database were computed. Through LASSO Cox regression analysis, genes to construct a risk model were identified. This method maintains the model’s predictive capability while reducing the number of variables through regularization to avoid overfitting. It involves adding a penalty term to the Cox model’s likelihood function, where the penalty is typically the sum of the absolute values of all coefficients multiplied by a tuning parameter λ. The selection of genes to be included in the final model is typically achieved through a variable selection process. This process involves using cross-validation to select an optimal λ value. For each λ, the model is estimated, and the λ that minimizes the cross-validation error is chosen for the model. Patients were classified into high-risk (≥ median risk score) and low-risk (< median risk score) categories. The survival outcomes of the two groups were compared using the Kaplan-Meier method and illustrated via survival curves. Receiver Operating Characteristic (ROC) curves were plotted to establish the model, with the Area Under the Curve (AUC) calculated to interpret predictive accuracy. Histograms predicting 1, 3, and 5-year survival rates were constructed using the rms and survival packages.

### Immune infiltration analysis

Different immune cells play distinct roles within the tumor microenvironment. To accurately assess the composition of immune cells in LIHC patient samples, we employed the CIBERSORT algorithm to examine the proportions of immune cells. Input files included expression data and a leukocyte signature matrix file (LM22.txt). Differences in the proportions of immune cells between high-risk and low-risk patients were compared using the Wilcoxon rank-sum test, with the statistical test results visualized through the R ggpubr package. Beyond immune cells, the tumor microenvironment also comprises tumor cells and stromal cells. The ESTIMATE package was utilized to calculate the tumor purity (ESTIMATEScore), stromal cell score (StromaScore), and immune score (ImmuneScore) for LIHC patients. The ggpubr and stats packages were used to explore the correlation between risk scores and the tumor immune microenvironment.

### Prediction of immune therapy response and drug sensitivity

We utilized the IMvigor210 cohort to assess the efficacy of anti-PD-L1 immune therapy, with a particular focus on investigating the predictive role of high-risk and low-risk patients in the context of immune checkpoint blockade (ICB) treatment. Using the chi-square test, we evaluated the differences in immune therapy response among patients with varying risk scores. Employing the Genomics of Drug Sensitivity in Cancer (GDSC) database, we predicted drug sensitivity for each sample using the R package “oncoPredict.” Additionally, we estimated the IC50 values for each sample and utilized the Wilcoxon rank-sum test to statistically analyze the differences in drug sensitivity between high-risk and low-risk patients.

### Cell culture

Human hepatoma cell lines (HepG2, Hep3B, Huh7, HCCLM3, PLC/PRF/5) and normal human hepatic cell lines (LO2, Chang liver, WRL68) were obtained from Zhongqiaoxinzhou Biotech (Shanghai, China). HepG2, Hep3B, Huh7, HCCLM3, Chang liver, and WRL68 were cultured in Dulbecco’s Modified Eagle Medium (DMEM) supplemented with 10% fetal bovine serum and 1% penicillin-streptomycin. The LO2 cell line was cultured in Roswell Park Memorial Institute 1640 medium (RPMI-1640) with the same supplements. Meanwhile, PLC/PRF/5 was cultured in Minimum Essential Medium (MEM) also supplemented with the same components. Cultures were maintained in a 37°C incubator with 5% CO2.

### Patient specimens

Human hepatocellular carcinoma and adjacent non-tumor tissues were obtained from surgical waste specimens at the Affiliated Hospital of Harbin Medical University. These tissues were subsequently preserved via cryopreservation and paraffin embedding. The study protocol was approved by the Medical Ethics Committee of the Second Affiliated Hospital of Harbin Medical University, with written informed consent obtained from the patients.

### RNA isolation and real-time quantitative reverse transcription polymerase chain reaction

Total RNA was extracted using Trizol reagent (Invitrogen, Carlsbad, CA, USA). The RNA was then reverse transcribed into cDNA using a reverse transcription kit (Toyobo, Japan). Quantitative assessment of mRNA levels was performed using SYBR Green Master Mix (Toyobo, Japan), with GAPDH or 18s serving as the internal control. Primers used are listed in **Supplementary Table S1**.

### Immunohistochemical staining

Paraffin sections of 5μm thickness were prepared, followed by deparaffinization and gradual rehydration through an ethanol gradient, and then thoroughly rinsed. Subsequently, the sections were mounted on poly-L-lysine-coated slides. Immunohistochemical staining was performed using multiple antibodies. A selection of primary antibodies was screened, including those against CLDN18 (#21126-1-AP, Proteintech, Wuhan, China, dilution 1:400) and GYPA (#bs-2575R, Bioss, Beijing, China, dilution 1:500).

### Transfection

siRNA was transfected into cells using jetPRIME^®^ transfection reagent (Polyplus, France). The sequences for human si-CLDN18 were as follows: si-CLDN18-#1 (target sequence: CAAGCACGACUAUGUGUAA); si-CLDN18-#3 (target sequence: CAGAAGAAACCAACUACAAAG).

### Scratch test and migration

Cells were cultured and transfected in serum-free medium within 6-well plates. Subsequently, wounds were created using the tip of a 10μl pipette, and changes in wound area from 0 to 24 hours were documented using a microscope (Olympus, Japan). In the transwell migration assay, treated cells were suspended in serum-free culture medium, and 200μL of the cell suspension was placed in the upper chamber. The lower chamber was filled with 500μL of culture medium containing 10% fetal bovine serum. After 24 hours, cells that had migrated to the lower surface were fixed with cold methanol and stained with crystal violet. Migration of the cells was recorded using a microscope (Olympus, Japan).

### Statistical analysis

Statistical analyses were primarily conducted using R software, version 4.1.0. Differential expressions were deemed statistically significant when the absolute value of the log fold change (FC) exceeded 2 and the *p*-value was less than 0.05. All experimental results were expressed as the mean ± standard error of the mean (SEM) of at least three independent experiments. Comparisons between two groups were conducted using Student’s *t*-test (two-tailed, unpaired), while comparisons among multiple groups were performed using one-way analysis of variance (ANOVA) followed by Dunnett’s multiple comparisons test. Data analysis was carried out using GraphPad Prism 9.0 software.

## Results

### Single-cell data processing

**Figure 1:**
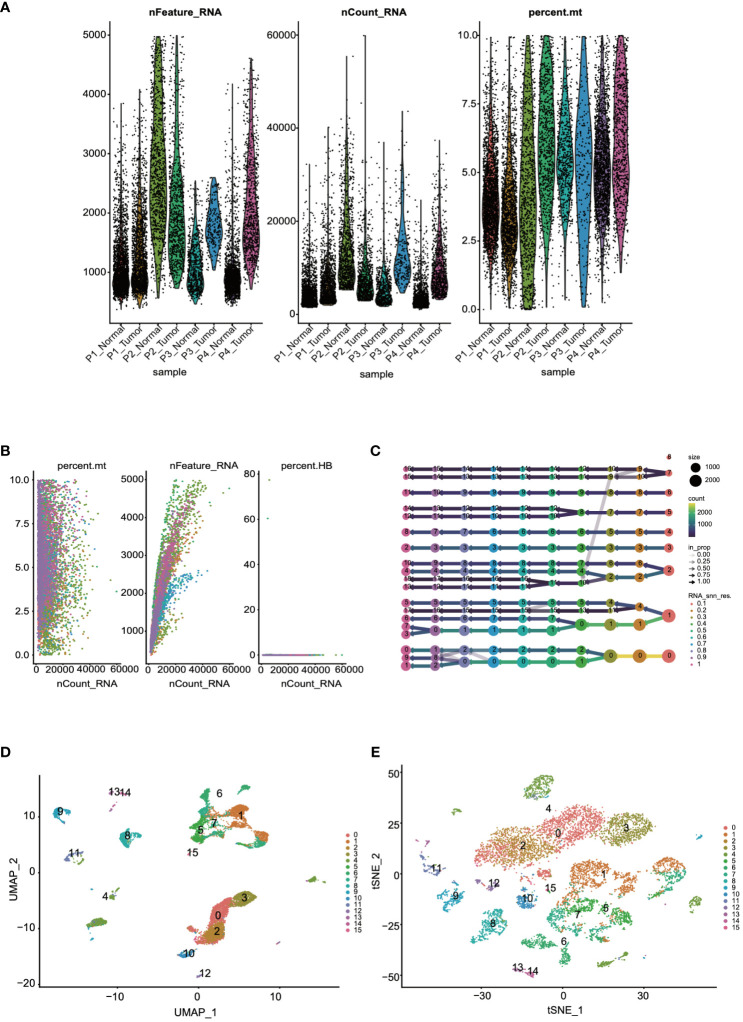
Quality control and visualization of single-cell data were conducted. **(A)** The dataset was filtered based on the number of genes and the percentage of mitochondrial genes. **(B)** An analysis of the correlations among RNA counts, mitochondrial content, and erythrocyte counts was performed. **(C)** Cells were clustered into groups based on their gene expression patterns. **(D, E)** The results of the clustering were visualized for further interpretation through UMAP and t-SNE.

Initially, to ensure the cells included in the dataset exhibited reasonable levels of gene expression, we filtered the dataset based on the number of genes and the percentage of mitochondrial gene expression. From the quality control charts, it was observed that by controlling the number of detected genes (> 200 and < 8000) and the percentage of mitochondrial gene expression (< 10%), we retained 60,288 cells (**Figure 1A**). Further analysis was conducted to explore cellular states and potential biological processes, revealing correlations between RNA counts, mitochondrial content, and erythrocyte counts (**Figure 1B**). For accurate clustering, we examined clustering results across a range of resolutions from 0.1 to 1, identifying the optimal clustering resolution at 0.5 (**Figure 1C**). Finally, to further validate the reliability and biological significance of the clustering, we visualized the clustering results using UMAP and t-SNE dimensionality reduction techniques (**Figures 1D, E**).

**Figure 2:**
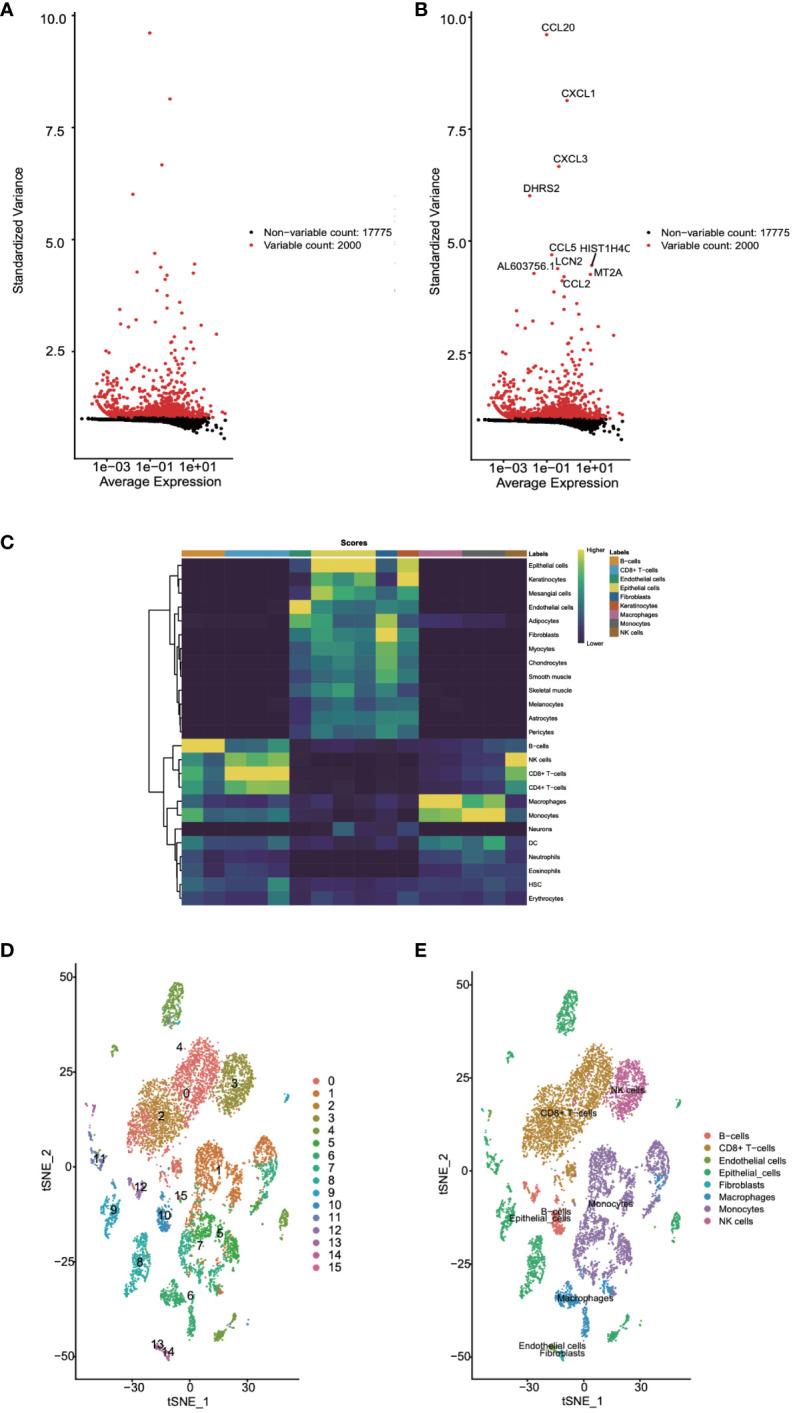
Cell annotation for single-cell data was undertaken. **(A, B)** Highly variable genes were identified. **(C)** The confidence levels of cell annotations were checked. **(D)** The distribution of cell types was visualized.

Next, we utilized the singleR package, an automated tool for cell type identification and annotation in single-cell RNA sequencing data, for cell annotation. Initially, we identified highly variable genes, which measure the variability among cells based on standard deviation. The variation of the top 2000 genes was found to represent the overall variability in the dataset (**Figures 2A, B**). Subsequently, we employed the plotScoreHeatmap function to display scores of all cells across all reference labels, examining the confidence of predicted labels across the dataset and presenting optimal annotation results (**Figure 2C**). We annotated a total of eight cell types: B cells, CD8+ T cells, endothelial cells, epithelial cells, fibroblasts, macrophages, monocytes, and NK cells. Furthermore, we visualized the distribution of cell types based on the t-SNE dimensionality reduction method (**Figures 2D**).

### Single-cell differential expression analysis

**Figure 3:**
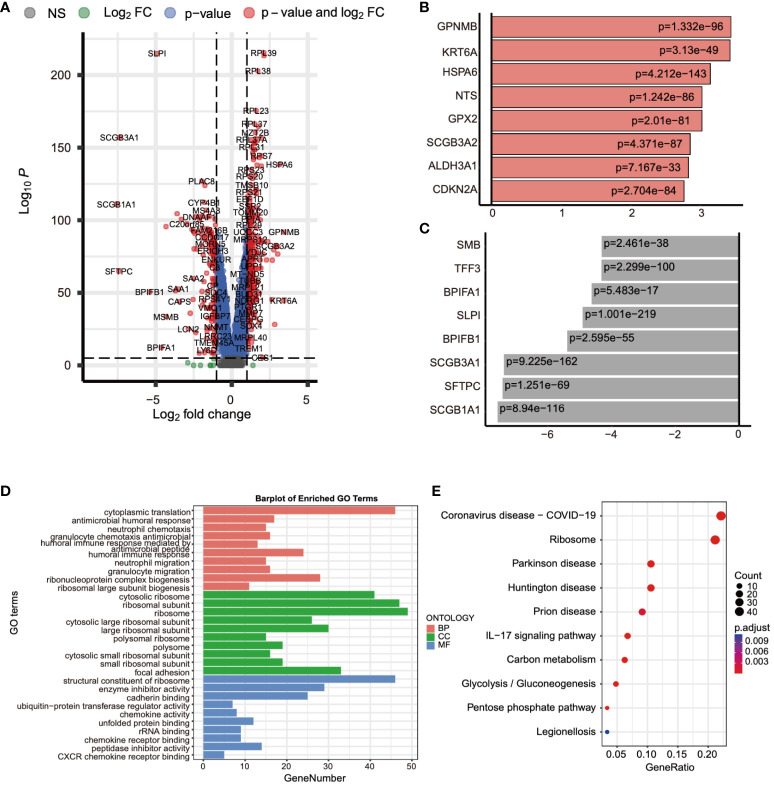
Differential expression analysis using single-cell data was performed. **(A)** The gene expression differences between normal and tumor tissues were compared using single-cell data. **(B)** The top eight genes with the most significant increases in expression were listed. **(C)** The top eight genes with the most significant decreases in expression were listed. **(D, E)** The biological functions and pathways involved with these differentially expressed genes were analyzed.

Following this, we leveraged single-cell data to conduct differential expression analysis between normal and tumor tissues. The analysis results, as depicted in **Figure 3A**, concurrently showcased the top 8 significantly upregulated genes (GPNMB, KRT6A, HSPA6, NTS, GPX2, SCGB3A2, ALDH3A1, CDKN2A) (**Figure 3B**) and the top 8 significantly downregulated genes (MSMB, TFF3, BPIFA1, SLPI, BPIFB1, SCGB3A1, SFTPC, SCGB1A1) (**Figure 3C**). Subsequent to this, we performed enrichment analysis on these differentially expressed genes, uncovering their involvement in biological processes such as granulocyte chemotaxis and the humoral immune response mediated by circulating immunoglobulins, as well as their association with chemokine-mediated molecular functions. Moreover, the IL-17 signaling pathway emerged as one of the significant pathways they partake in (**Figures 3D, E**). These findings contribute to a deeper understanding of the gene expression differences between normal and tumor tissues, thereby revealing potential biological processes and pathways associated with tumor pathogenesis and progression.

### Analysis of genes associated with ADCP

**Figure 4:**
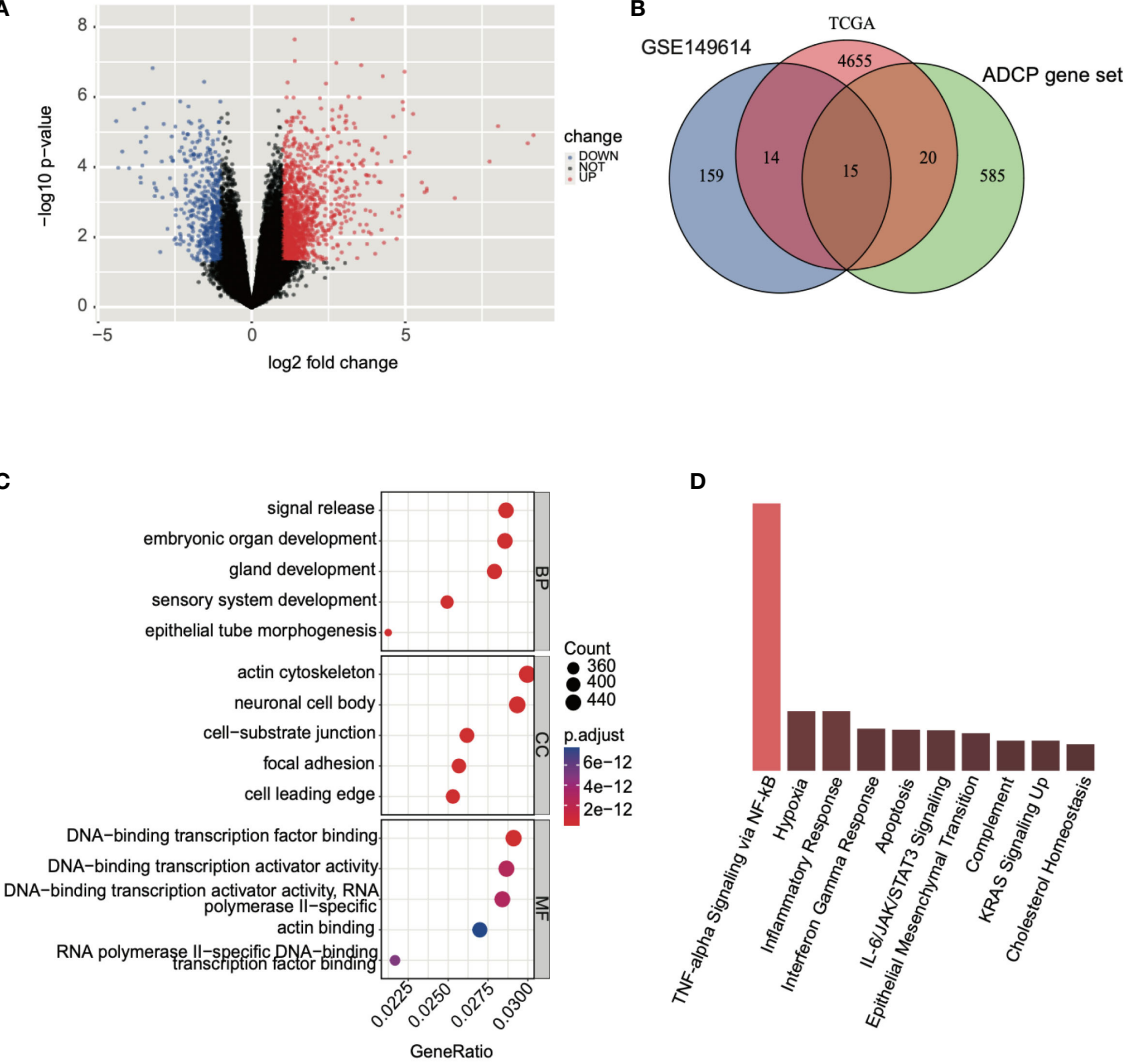
Analysis of genes associated with ADCP was conducted. **(A)** Differential expression analysis was performed on TCGA-LIHC expression profile data, where blue represented downregulated genes and red indicated upregulated genes. **(B)** An intersection was made between the results of the single-cell data differential analysis, TCGA differential analysis, and the ADCP gene set. **(C, D)** GO and KEGG analyses were conducted on the intersected genes.

Subsequently, we conducted a differential expression analysis on TCGA expression profile data, identifying a total of 4,704 differentially expressed genes (2,139 upregulated and 2,565 downregulated) and illustrated the results in a volcano plot (**Figure 4A**). Following this, we intersected the differential analysis results from single-cell data, TCGA differential analysis, and the ADCP gene set, yielding 15 ADCP-related genes (**Figure 4B**). Further analysis of these genes using GO (Gene Ontology) and KEGG (Kyoto Encyclopedia of Genes and Genomes) revealed that the intersected genes were involved in NF-κB-mediated TNF-α signaling, the interferon-gamma (IFN-γ) response in inflammation, and the IL-6/JAK/STAT3 signaling pathway (**Figures 4C, D**).

### Construction of a prognostic model based on ADCP-related genes

**Figure 5:**
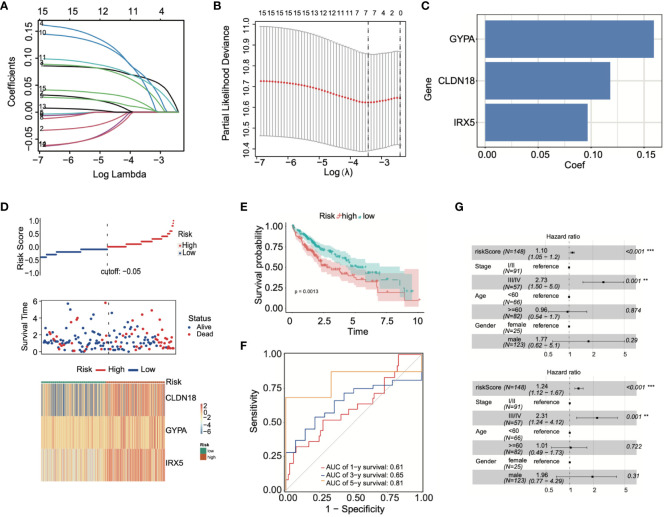
Prognostic model was built using ADCP-related genes. **(A, B)** LASSO Cox regression analysis was conducted using TCGA-LIHC data. **(C)** Patients’ risk scores were calculated based on three prognostic genes. **(D)** The correlation between risk scores and survival status was analyzed. **(E)** Differences in survival between high-risk and low-risk groups were examined through Kaplan-Meier survival curve analysis. **(F)** The predictive capability of the model was demonstrated through ROC curve analysis. **(G)** The effectiveness of the model in prognostic evaluation was determined through univariate and multivariate Cox analysis. **p < 0.01; and ***p < 0.001.

By conducting LASSO Cox regression analysis within the TCGA database, we identified three genes (GYPA, CLDN18, and IRX5) for the establishment of a risk model (**Figures 5A, B**). The risk score was calculated using the following formula, based on the coefficients of these genes: Risk Score = GYPA × (0.159) + CLDN18 × (0.118) + IRX5 × (0.096) (**Figure 5C**). This formula was employed to compute the risk score for each patient. Subsequently, LIHC patients were divided into high-risk and low-risk groups based on the median risk score. **Figure 5D** displayed the distribution of risk scores and survival status. As shown in **Figure 5E**, the overall survival (OS) of high-risk patients was significantly worse than that of low-risk patients, as determined by Kaplan-Meier survival curve analysis (*p* = 0.0013). Following this, 1-year, 3-year, and 5-year ROC curves were generated, demonstrating the model’s robust predictive capability (**Figure 5F**). According to univariate and multivariate Cox analyses, the risk model served as a crucial indicator for assessing the prognosis of LIHC patients (**Figure 5G**).

### Immune infiltration analysis

**Figure 6:**
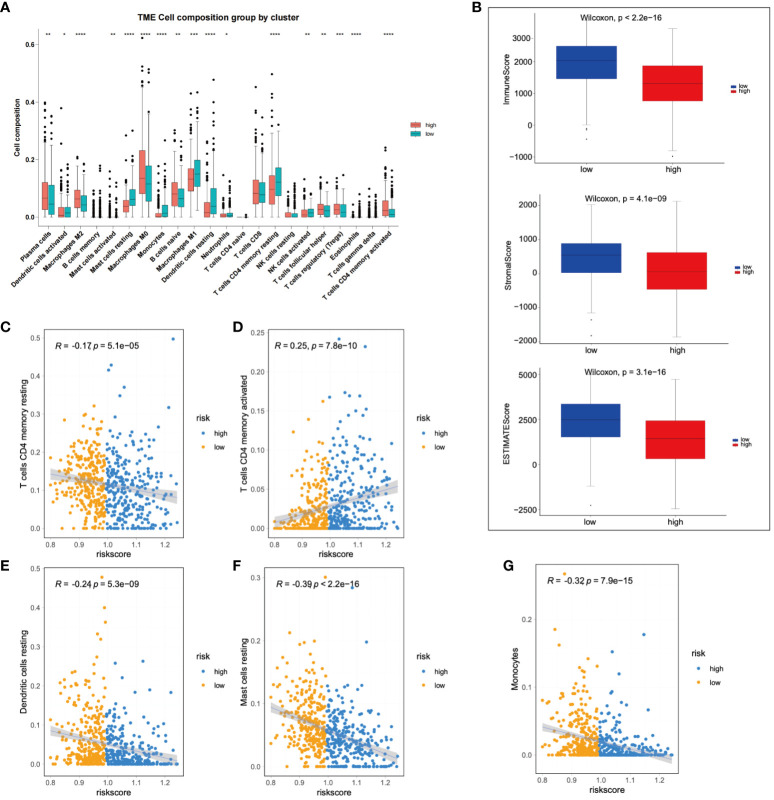
Immunological assessment of high-risk and low-risk groups was performed. **(A)** A comparison of changes in 22 types of immune cells was made between high-risk and low-risk group patients. **(B)** A comparison of stromal, immune, and ESTIMATE scores was conducted between high-risk and low-risk patients. **(C-G)** The correlation between immune infiltrating cells and risk scores was analyzed. *p < 0.05; **p < 0.01; and ***p < 0.001.

Subsequent to this, we compared the changes in 22 types of immune cells between high-risk and low-risk patients, finding significant enrichment of M2 macrophages in the high-risk group. Conversely, neutrophils, dendritic cells (DCs) and M1 macrophages were significantly enriched in low-risk patients (**Figure 6A**). Further, the immune status of LIHC patients was analyzed based on the prognostic model. The ESTIMATE algorithm was used to calculate stromal, immune, and ESTIMATE scores for low-risk and high-risk patients, respectively. The results demonstrated significant differences in stromal and ESTIMATE scores between low-risk and high-risk patients (**Figure 6B**). Lastly, immune infiltrating cells were examined to determine their correlation with risk scores. A positive correlation was observed between activated memory CD4+ T cells and risk scores, while monocytes, resting mast cells, resting memory CD4+ T cells, and resting dendritic cells showed a negative correlation with risk scores (**Figures 6C-G**).

### Application of prognostic models

**Figure 7:**
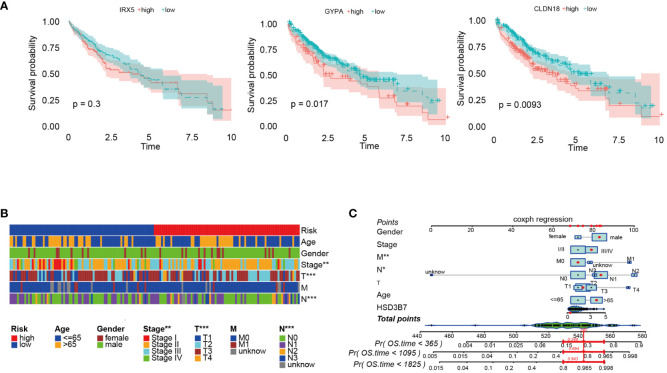
Further analysis of the prognostic model was conducted. **(A)** Survival analysis was performed on three prognostic genes. **(B)** Survival rates of high-risk and low-risk patients within various clinical subgroups were compared. **(C)** The prognostic risk model was analyzed using nomogram. *p < 0.05; **p < 0.01; and ***p < 0.001.

Initially, we analyzed the association between three key genes and survival in high- and low-risk groups, finding that GYPA and CLDN18 acted as risk factors, while IRX5 showed no significant relation to survival (**Figure 7A**). To assess the accuracy of the prognostic model, patients were subgrouped based on vital clinical indicators such as age, gender, and clinical staging. Survival rate analysis was conducted by comparing high-risk and low-risk patients within each subgroup. The results indicated that, across different subgroups considering age, gender, and tumor TNM staging, low-risk patients exhibited significantly better overall survival rates than high-risk patients (*p*-value < 0.05), thereby validating the prognostic model as an independent predictor of patient prognosis (**Figure 7B**). Furthermore, nomogram was created to facilitate the application of this model in clinical practice (**Figure 7C**).

**Figure 8:**
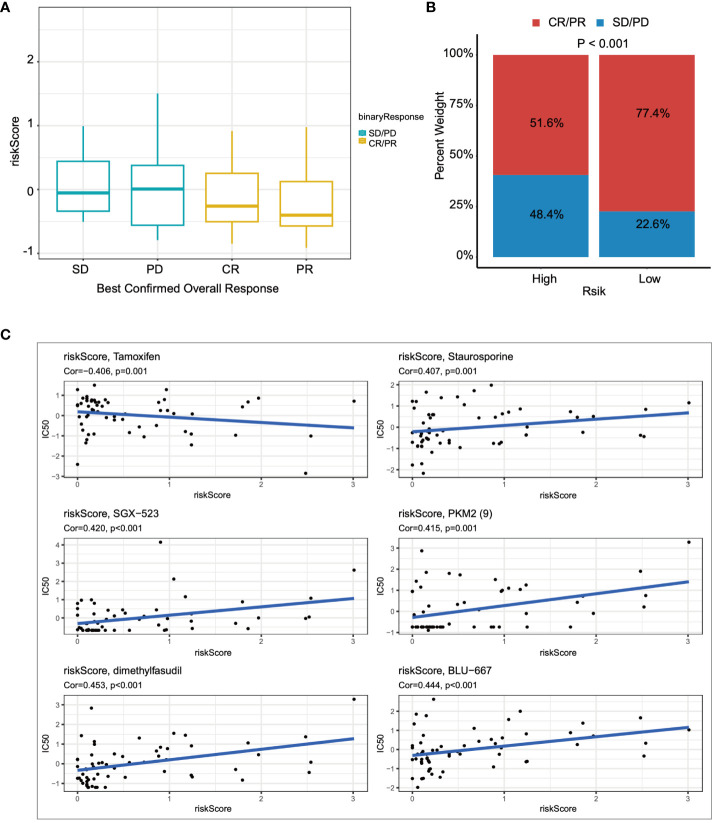
The relationship between the prognostic model and immunotherapy was evaluated. **(A)** The relationship between risk scores and outcomes of immunotherapy was assessed. **(B)** The proportions of CR/PR in high-risk and low-risk patients were compared. **(C)** The association between risk scores and drug sensitivity was evaluated through Spearman correlation analysis.

Lastly, we utilized the IMvigor210 immunotherapy cohort to assess the relationship between risk scores and outcomes of immunotherapy. Patients achieving partial response (PR) or complete response (CR) exhibited lower risk scores compared to those with stable disease (SD) or disease progression (PD) (**Figure 8A**). Furthermore, the proportion of patients achieving CR/PR was significantly lower in the high-risk group compared to those with low risk scores (*p* < 0.001) (**Figure 8B**). Finally, drugs closely associated with the risk scores were identified through Spearman correlation analysis. The results demonstrated a significant association between risk scores and six drugs in the GDSC database, including Tamoxifen, Staurosporine, and SGX−523, among others (**Figure 8C**).

### Pan-cancer analysis of CLDN18

**Figure 9:**
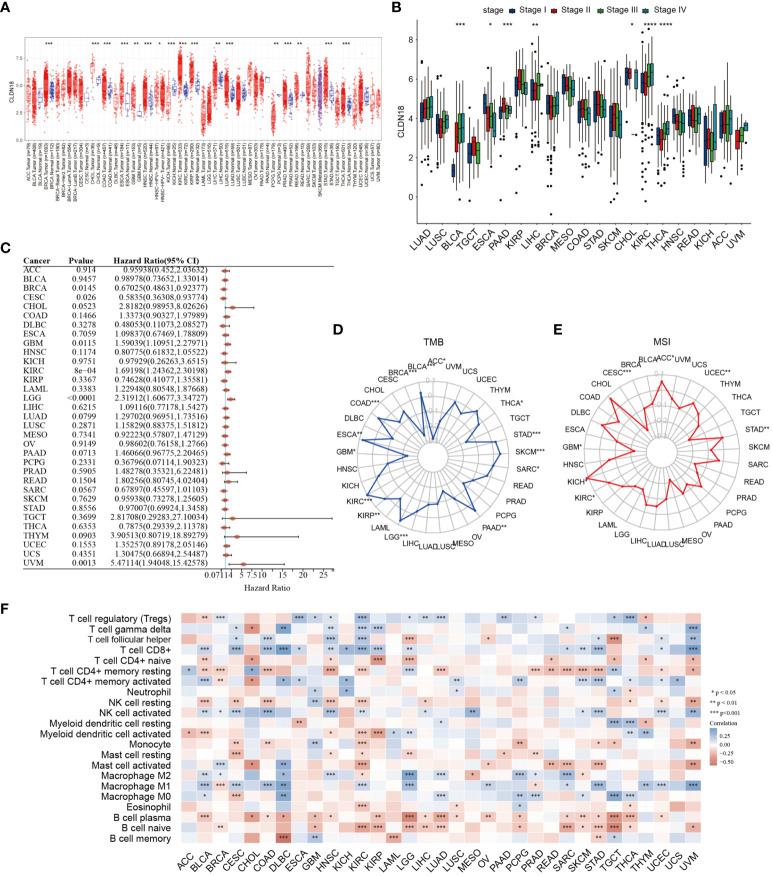
A pan-cancer analysis of CLDN18 was carried out. **(A)** The expression levels of CLDN18 across various cancers were analyzed. **(B)** The correlation between CLDN18 expression and cancer progression was assessed. **(C)** The association between CLDN18 expression and cancer prognosis was evaluated. **(D)** The relationship between CLDN18 expression and cancer TMB was analyzed. **(E)** The association between CLDN18 expression and cancer MSI was evaluated. **(F)** The correlation between CLDN18 expression and cancer immune infiltration was assessed. *p < 0.05; **p < 0.01; and ***p < 0.001.

Subsequently, we investigated the expression levels of CLDN18 across various cancer types and found that CLDN18 was highly expressed in multiple cancer types (**Figure 9A**). Moreover, we observed an increase in CLDN18 expression levels with the progression of several cancer types (e.g., LIHC, KIRC) (**Figure 9B**). A forest plot also revealed that CLDN18 was associated with the prognosis of multiple cancers (**Figure 9C**). We examined the relationship between CLDN18 expression levels and MSI (Microsatellite Instability) and TMB (Tumor Mutation Burden) to determine if CLDN18 could serve as a predictive marker for the response to immunotherapy across various cancer types. CLDN18 expression was positively correlated with TMB in ESCA, STAD, SKCM, and KIRC, and negatively correlated with TMB in PRAD, DLBC, READ, and BRCA (**Figure 9D**). Additionally, CLDN18 expression was positively correlated with MSI in ACC, KICH, and STAD, and negatively correlated with MSI in CESC, GBM, and TGCT (**Figure 9E**). Immune infiltration analysis revealed correlations between CLDN18 expression and various types of immune cells in several cancer types, including LIHC (**Figure 9F**).

### Experimental validation of GYPA and CLDN18

**Figure 10:**
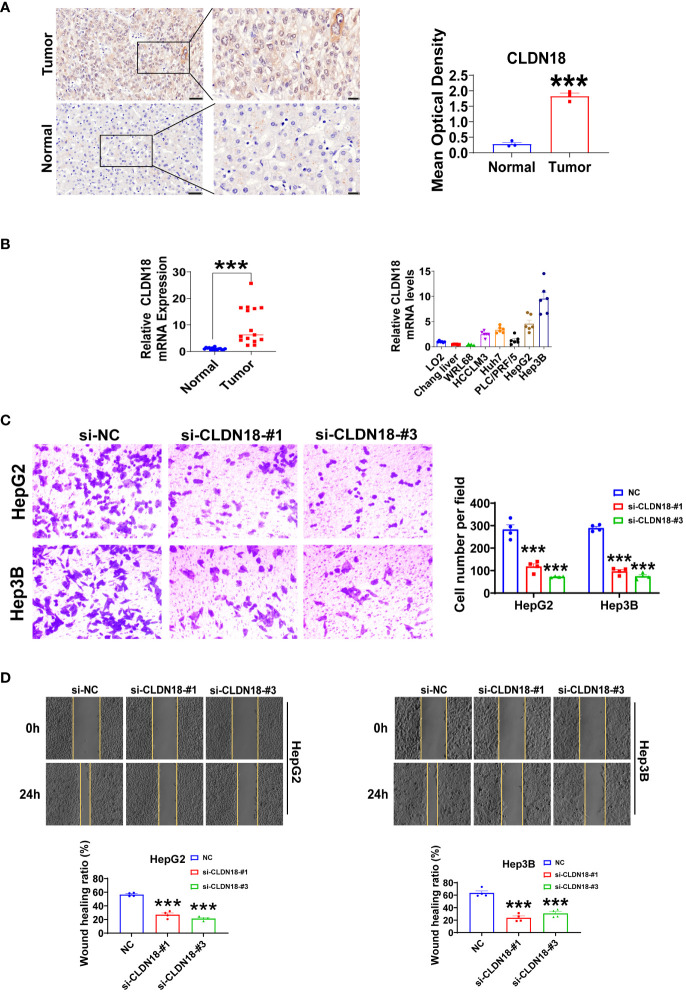
Experiments were conducted to validate CLDN18. **(A)** Immunohistochemical staining was utilized to analyze the expression levels of CLDN18 in hepatocellular carcinoma and adjacent non-tumor tissues. The scale bars in the stained tissue images measured 50 μm, 20 μm (*n* = 3). **(B)** The expression levels of CLDN18 in hepatocellular carcinoma, adjacent non-tumor tissues (*n* = 15), normal hepatic cell lines, and hepatocellular carcinoma cell lines (*n* = 6) were analyzed using RT-qPCR experiments. **(C)** Transwell assays were conducted to investigate the effects of CLDN18 knockdown on the migratory capabilities of HepG2 and Hep3B cells *in vitro* (*n* = 4). **(D)** Scratch assays were performed to explore the impact of CLDN18 knockdown on the migration abilities of HepG2 and Hep3B cells *in vitro* (*n* = 4). **p < 0.01; and ***p < 0.001.

We initially compared the expression differences of GYPA and CLDN18 between human hepatocellular carcinoma and adjacent non-tumor tissues using immunohistochemistry staining. We observed a significant upregulation of GYPA and CLDN18 in hepatocellular carcinoma tissues (**Figure 10A**; **Supplementary Figure S1A**). Subsequently, we assessed the differences in mRNA expression levels of GYPA and CLDN18 between human hepatocellular carcinoma and adjacent non-tumor tissues, as well as between normal liver cell lines and hepatocellular carcinoma cell lines. Our findings indicated an upregulation of GYPA and CLDN18 mRNA levels in hepatocellular carcinoma tissues, with CLDN18 expression elevated in hepatocellular carcinoma cell lines and GYPA expression increased in most hepatocellular carcinoma cell lines (**Figure 10B**; **Supplementary Figure S1B**). Following this, we investigated the impact of CLDN18 expression on cell migration. Scratch assays and Transwell migration experiments demonstrated that knockdown of CLDN18 significantly inhibited the migration of HepG2 and Hep3B cells (**Figures 10C, D**). And the validation of knockdown efficiency for CLDN18 was shown in **Supplementary Figure S1C**.

## Discussion

To date, only a handful of studies have designed experiments to explore the role of ADCP in LIHC, focusing on targets that are relatively singular and nonspecific (13, 14). Our study represents the first comprehensive report to analyze the regulation of genes involved in ADCP within the context of LIHC. Building upon the foundation laid by the research of Kamber RA et al., which identified genes associated with ADCP, our study employed bioinformatics and corroborative experimental validation to sift through these genes, identifying those with significant prognostic value and immune sensitivity as ADCP-related biomarkers. We discovered that GYPA, CLDN18, and IRX5 could be pivotal genes regulating ADCP in LIHC. These genes showed a promising correlation with the efficacy of immunotherapy in LIHC and were closely linked to clinical pathological staging and patient prognosis. Within our risk model, the prognostically significant genes GYPA and CLDN18 were validated using clinical samples. Furthermore, CLDN18 was subjected to pan-cancer analysis and related *in vitro* regulatory experiments, establishing its central targeting role.

To begin, we utilized single-cell data to conduct a differential expression analysis between normal and LIHC tumor tissues, identifying several top upregulated and downregulated genes that had been reported in tumor immunity. These differential genes were associated with granulocyte chemotaxis and migration, humoral immune responses mediated by circulating immunoglobulins, chemokines, and participated in the IL-17 signaling pathway. Neutrophils, crucial innate immune cells, play a key role in various diseases, including cancer (15). Bispecific antibody therapy could recruit cell types, including macrophages and neutrophils, as effector cells in cancer immunotherapy to induce ADCP (16). The novel recombinant SIRPα-Fc fusion protein IMM01 could activate macrophages during the ADCP induction process. Activated macrophages exert anti-tumor effects by increasing immune cell infiltration through the secretion of chemokines (17). IL-17, a pro-inflammatory cytokine produced by a specialized group of T helper cells known as Th17 cells, operates through a signaling pathway independent from ADCP. However, IL-17 could alter the local environment by promoting the production of inflammatory mediators, potentially enhancing the recruitment and activation of phagocytic cells, thereby augmenting the effects of ADCP (18). Therefore, our findings underscored the pivotal role of ADCP in the pathophysiology of LIHC. Furthermore, in colorectal cancer with amplified RBP4+NTS+ cancer cell subpopulations, macrophage-induced ADCP is more pronounced and correlates with a favorable prognosis (19). NTS was among the top differential genes we identified.

Similarly, the ADCP-related genes identified in our LIHC study were involved in the TNF-α signaling pathway of NF-κB, the IFN-γ response in inflammatory reactions, and the IL-6/JAK/STAT3 signaling pathway. Besla R et al. discovered that T-cell dependent bispecific antibodies could activate NK cells, enhancing their antibody-dependent cellular cytotoxicity, while also increasing the ability of macrophages to execute ADCP. This enhancement was triggered by cytokines released during antibody therapy, with IFN-γ being the primary driver for ADCP enhancement, and TNFα further augmenting the cytotoxic capability of macrophages (20). Through LASSO Cox regression analysis, we identified three genes (GYPA, CLDN18, and IRX5) to construct a risk model. To date, there have been no reports on the role of GYPA in LIHC and ADCP. IRX5 has also not been explored in the context of ADCP. Current research suggested IRX5 was a potential downstream target of miR-136-5P, which could increase the tumorigenicity of LIHC cells (21). Additionally, IRX5 could inhibit apoptosis in HCC cells by suppressing the p53 signaling pathway (22). Whether its roles in tumorigenicity or anti-apoptosis are related to ADCP remains unknown, and further investigation is needed to determine if IRX5 enhances the malignant capabilities of liver cancer cells by conferring resistance to ADCP. CLDN18 has been identified as a potential prognostic marker and immunotherapeutic target in LIHC (23). Furthermore, researchers have developed a novel bispecific antibody, PT886, targeting CLDN18.2 and CD47. CLDN18.2 is overexpressed in the majority of gastric adenocarcinomas and pancreatic cancers. Antibodies targeting CLDN18.2 could redirect macrophage-mediated phagocytic activity towards tumor cells, thereby enhancing anti-tumor activity (24). Our study also confirmed the role of CLDN18 in regulating ADCP in LIHC, underscoring the validity and potential translational value of our research. We hypothesized that the tumor microenvironment might induce glycosylation changes in GYPA, interfering with the antibody binding sites and thereby reducing the efficiency of ADCP. Furthermore, the overexpression of CLDN18 could enhance the barrier function of tumor cells, obstructing macrophage contact with and recognition of tumor cells. In the risk scoring model, patients in the low-risk group exhibited better prognoses, characterized by a higher infiltration of neutrophils, M1-type macrophages, and dendritic cells, and a reduced infiltration of M2-type macrophages. Neutrophils are specialized phagocytes that protect the host from infections. In oncology research, rituximab has been shown to induce neutrophil-mediated phagocytosis of B-cell lymphoma cells. Indeed, neutrophil-mediated ADCP has been reported in various monoclonal antibody therapies, including obinutuzumab, ofatumumab, and trastuzumab (15). Macrophages, a crucial component of the innate immune system, are broadly categorized into two subtypes in the tumor microenvironment: the tumor-suppressing M1 type and the tumor-promoting M2 type. Li H and colleagues found that M1-type macrophages elicited a more effective ADCP response than M2 types (25). Similarly, Yan M et al. discovered that FcγR-dependent M1-type macrophage-mediated ADCP was essential for maintaining anti-lymphoma activity, suggesting that strategies promoting the recruitment of M1-type macrophages or repolarization of macrophages could enhance the response to immunotherapy in lymphoma (26). This aligns with the trends observed in our liver cancer study. Therefore, we believe that the infiltration of neutrophils, M1-type macrophages, and dendritic cells is abundant in patients with low-risk, and this is directly related to their better prognosis. Our immunoscore and assessment of immunotherapy response further validated the accuracy of our risk scoring model. A pan-cancer analysis of our core target, CLDN18, revealed significant associations with prognosis and immunity across various cancers, confirming its potential for broad application. Further experimental validation in tissue and cell samples demonstrated that CLDN18 was upregulated in cancer tissues and cells, and knocking down CLDN18 inhibited the malignant behavior of liver cancer cells. This suggests that CLDN18, as an immunological target, may not only confer resistance to ADCP in liver cancer cells but also enhance their malignancy. In summary, the prognostic and therapeutic value of CLDN18 in LIHC warrants further investigation.

Moreover, our study is not without its limitations. Firstly, the results of our analysis should be validated through more comprehensive clinical staging. The role of targets in ADCP within LIHC requires further in-depth *in vitro* and *in vivo* investigation. Secondly, although we have explored the correlation between the risk model and therapeutic drugs, we have not delved into the potential mechanisms of action of these drugs in ADCP. Additionally, despite our comprehensive pan-cancer analysis, the generalizability of our findings still necessitates further evidential support.

## Conclusion

Through the integration of scRNA-seq and Bulk RNA-seq data in hepatocellular carcinoma, we have identified ADCP regulatory factors, uncovering an ADCP signature comprising three genes associated with LIHC immunity and prognosis. Analysis based on a risk scoring model derived from these three genes, followed by experimental validation, confirmed the pivotal role of M1-type macrophages in ADCP within LIHC, with CLDN18 being identified as a crucial ADCP regulatory target in LIHC.

## Data availability statement

The datasets presented in this study can be found in online repositories. The names of the repository/repositories and accession number(s) can be found in the article/**Supplementary Material**.

## Ethics statement

The studies involving humans were approved by the Medical Ethics Committee of the Second Affiliated Hospital of Harbin Medical University. The studies were conducted in accordance with the local legislation and institutional requirements. The participants provided their written informed consent to participate in this study.

## Author contributions

ZZ: Conceptualization, Data curation, Formal analysis, Methodology, Project administration, Writing – original draft, Writing – review & editing. YuL: Formal analysis, Methodology, Validation, Writing – review & editing. ZQ: Formal analysis, Methodology, Software, Writing – review & editing. YaL: Formal analysis, Investigation, Software, Writing – review & editing. LZ: Resources, Validation, Visualization, Writing – review & editing. SS: Project administration, Resources, Supervision, Validation, Writing – review & editing. XC: Formal analysis, Investigation, Resources, Validation, Visualization, Writing – review & editing.

## Supplementary material

Supplementary Figure 1 Experiments were conducted to validate GYPA and CLDN18. **(A)** Immunohistochemical staining was employed to examine the expression levels of GYPA in hepatocellular carcinoma and surrounding non-tumorous tissues. The scale bars in the stained tissue images measured 50 μm, 20 μm (*n* = 3). **(B)** The expression levels of GYPA in hepatocellular carcinoma, adjacent non-tumor tissues (*n* = 15), normal hepatic cell lines, and hepatocellular carcinoma cell lines (*n* = 6) were analyzed using RT-qPCR experiments. **(C)** The knockdown efficiency of CLDN18 was validated in the HepG2 and Hep3B cells (*n* = 4). ***p* < 0.01; and ****p* < 0.001.
