# Supplementary figures and images for: Constructing immune and prognostic features associated with ADCP in hepatocellular carcinoma and pan-cancer based on scRNA-seq and bulk RNA-seq
Source: Front Immunol. 2024 May 7;15:1397541. doi: 10.3389/fimmu.2024.1397541 (PMC11106372; doi:10.3389/fimmu.2024.1397541)

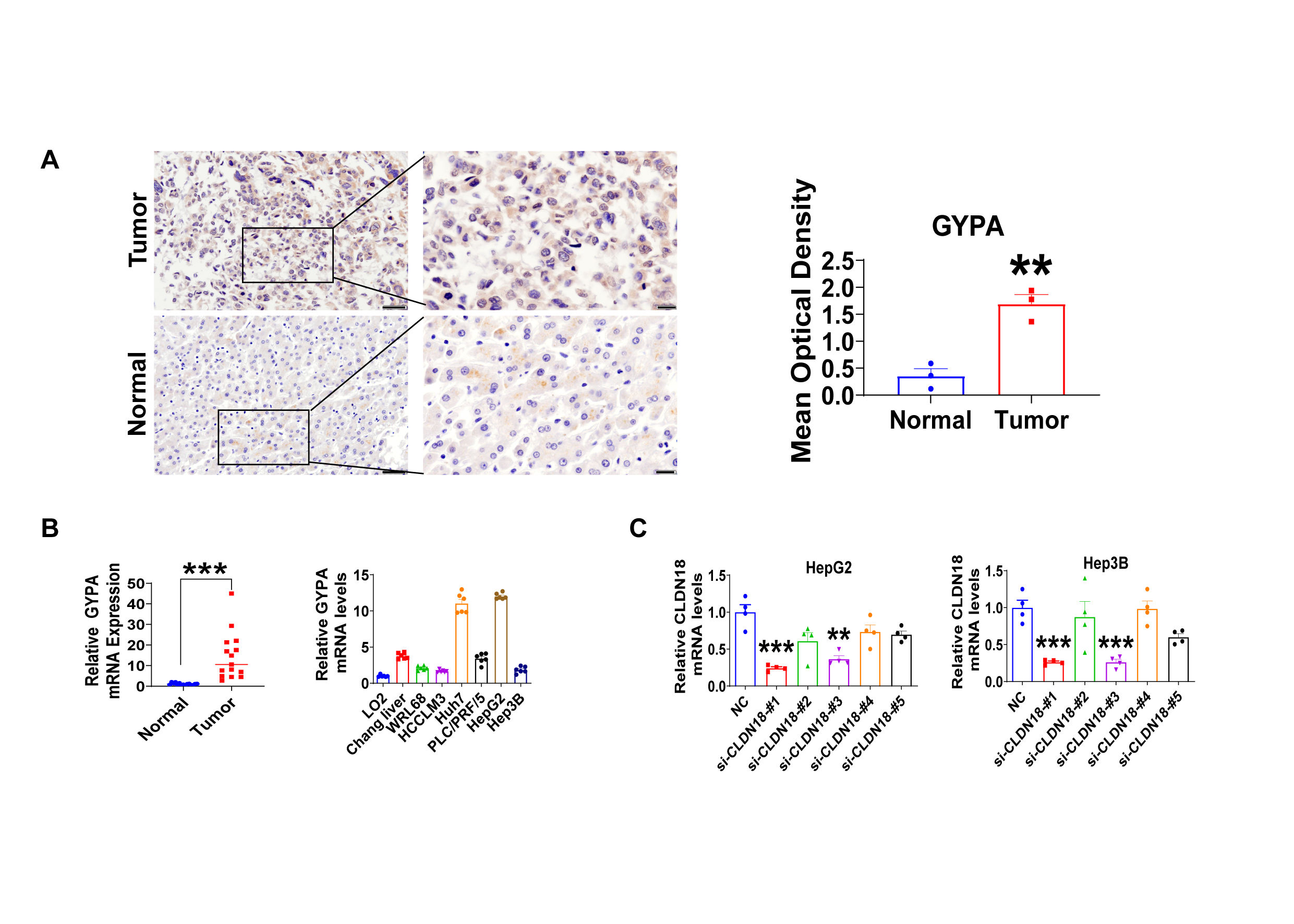

Supplement: Supplementary Figure 1 — Experiments were conducted to validate GYPA and CLDN18. (A) Immunohistochemical staining was employed to examine the expression levels of GYPA in hepatocellular carcinoma and surrounding non-tumorous tissues. The scale bars in the stained tissue images measured 50 μm, 20 μm (n = 3). (B) The expression levels of GYPA in hepatocellular carcinoma, adjacent non-tumor tissues (n = 15), normal hepatic cell lines, and hepatocellular carcinoma cell lines (n = 6) were analyzed using RT-qPCR experiments. (C) The knockdown efficiency of CLDN18 was validated in the HepG2 and Hep3B cells (n = 4). **p < 0.01; and ***p < 0.001. [file Image_1.tif]
